# Intraganglionic AAV6 Results in Efficient and Long-Term Gene Transfer to Peripheral Sensory Nervous System in Adult Rats

**DOI:** 10.1371/journal.pone.0061266

**Published:** 2013-04-16

**Authors:** Hongwei Yu, Gregory Fischer, Lejla Ferhatovic, Fan Fan, Alan R. Light, Dorothee Weihrauch, Damir Sapunar, Hiroyuki Nakai, Frank Park, Quinn H. Hogan

**Affiliations:** 1 Department of Anesthesiology, Medical College of Wisconsin, Milwaukee, Wisconsin, United States of America; 2 Department of Anatomy, Histology and Embryology, University of Split School of Medicine, Split, Croatia; 3 Department of Pharmacology and Toxicology, Mississippi University Medical Center, Jackson, Mississippi, United States of America; 4 Departments of Anesthesiology, Neurobiology and Anatomy, University of Utah, Salt Lake City, Utah, United States of America; 5 Molecular and Medical Genetics, Oregon Health and Science University, Portland, Oregon, United States of America; 6 Department of Physiology and Medicine, Medical College of Wisconsin, Milwaukee, Wisconsin, United States of America; 7 Zablocki Veterans Affairs Medical Center, Milwaukee, Wisconsin, United States of America; Justus-Liebig-University Giessen, Germany

## Abstract

We previously demonstrated safe and reliable gene transfer to the dorsal root ganglion (DRG) using a direct microinjection procedure to deliver recombinant adeno-associated virus (AAV) vector. In this study, we proceed to compare the *in vivo* transduction patterns of self-complementary (sc) AAV6 and AAV8 in the peripheral sensory pathway. A single, direct microinjection of either AAV6 or AAV8 expressing EGFP, at the adjusted titer of 2×10^9^ viral particle per DRG, into the lumbar (L) 4 and L5 DRGs of adult rats resulted in efficient EGFP expression (48±20% for AAV6 and 25±4% for AAV8, mean ± SD) selectively in sensory neurons and their axonal projections 3 weeks after injection, which remained stable for up to 3 months. AAV6 efficiently transfers EGFP to all neuronal size groups without differential neurotropism, while AAV8 predominantly targets large-sized neurons. Neurons transduced with AAV6 penetrate into the spinal dorsal horn (DH) and terminate predominantly in superficial DH laminae, as well as in the dorsal columns and deeper laminae III-V. Only few AAV8-transduced afferents were evident in the superficial laminae, and spinal EGFP was mostly present in the deeper dorsal horn (lamina III-V) and dorsal columns, with substantial projections to the ventral horn. AAV6-mediated EGFP-positive nerve fibers were widely observed in the medial plantar skin of ipsilateral hindpaws. No apparent inflammation, tissue damage, or major pain behaviors were observed for either AAV serotype. Taken together, both AAV6 and AAV8 are efficient and safe vectors for transgene delivery to primary sensory neurons, but they exhibit distinct functional features. Intraganglionic delivery of AAV6 is more uniform and efficient compared to AAV8 in gene transfer to peripheral sensory neurons and their axonal processes.

## Introduction

Chronic pain, such as that which follows nerve injury, is common and inadequately treated. Drug development for the treatment of chronic neuropathic pain has focused on agents that target specific biomolecules of interest in the sensory pathway. Although many biological and pharmacological molecules have potential to modulate sensory neuron function in chronic pain models, there are major problems in delivering these agents into the relevant cell populations and sites. Disordered cellular mechanisms underlying chronic pain after peripheral nerve injury reside at diverse sites, including in receptive fields in peripheral tissues, in the somata of the injured sensory neurons, and in the dorsal horn (DH) of the spinal cord [1]. The dorsal root ganglia (DRGs), which harbor the somata of primary sensory neurons, are thus optimally situated as sites for pain pharmacotherapy. Direct injection into the DRG is well tolerated in both human and rodent subjects [2], [3], [4], [5].

DRG-targeted gene delivery is a potential therapeutic option for reversing neuronal pathology in neuropathic pain. To date, the most successful gene therapy strategies rely on recombinant viral vectors (e.g. adeno-associated virus, adenovirus, lentivirus, and retrovirus), although the utility of non-viral vectors is continuing to emerge [6]. Enthusiasm for the recombinant adeno-associated virus (AAV) vector system for *in vivo* viral gene transfer has grown in recent years. Despite the small transgene-packaging capacity of AAV, this vector offers the advantages of an ability to transduce post-mitotic cells (including primary sensory neurons), relatively high efficiency in transduction, long-term episomal expression, and replication deficiency [7], [8], [9]. Moreover, AAV vectors exhibit minimal immunogenicity and have a limited ability to transduce antigen-presenting cells, such as dendritic cells and macrophages [10]. Importantly, AAV has not been associated with any direct human pathogenesis, making it a desirable gene delivery system for clinical applications.

Studies have reported high efficacy and safety of recombinant AAV as a vector for gene delivery to primary sensory pathways [2], [3], [11], [12], [13]. Early studies showed that intraganglionic or intrasciatic nerve delivery of prototypic AAV2 (vector packaging AAV2 recombinant genomes with serotype 2 capsid) exhibits neuronal transduction in the DRG [14], [15], [16], [17]. Since the isolation of AAV2, other novel naturally occurring serotypes and numerous variants of AAV have been identified by viral capsid protein sequences, which varies among serotypes [18], [19]. In recent years, recombinant AAV vectors based on these novel serotypes have been explored for better gene transfer performance in peripheral sensory systems, including AAV1, AAV5, AAV6, AAV8 and AAV9 by various delivery strategies [2], [3], [5], [11], [13], [20], [21], [22], [23], [24], [25]. *In vivo* application of various AAV vectors consistently show neuronal tropism in the DRG [2], [7], [26], although the particular AAV serotype strongly influences the pattern of transduction for specific DRG neuronal subpopulations. These encouraging initial results indicate that AAV-based *in vivo* gene delivery to DRG neurons may be developed as a versatile experimental manipulation for pain research, and as a possible therapeutic approach [7]. However, there have been only limited direct comparisons of different AAV vectors for direct DRG injection.

We have previously reported that intraganglionic AAV8 is an efficient vector to deliver transgenes preferentially to large-sized DRG neurons with an early onset and a safe profile [2]. In the present study, we have extended our investigations in an effort to test if AAV6 can enhance DRG transduction in small-sized nociceptive neuron population, in comparison to AAV8. Since variations in the specific techniques used to generate vectors can substantially affect their efficacy and toxicity [27], [28], [29], our experimental design employs a direct comparison of AAV8 and AAV6 with tight controls to assure comparable vector preparations. We report here that microinjection of AAV6 into lumbar DRGs results in more efficient gene transfer to the nociceptive DRG neuron population when compared to AAV8, resulting in sustained and innocuous transgene expression that extends to the central and peripheral axonal processes and terminals of transduced neurons.

## Materials and Methods

### Ethics Statement

This study was carried out in strict accordance with the recommendations in the Guide for the Care and Use of Laboratory Animals of the National Institutes of Health. All animal procedures were approved by the Zablocki VA Medical Center Animal Studies Subcommittee and Medical College of Wisconsin IACUC (Permit number: 3690-03).

### Animals

Male Sprague Dawley (SD) rats (5–6 weeks old; 125–150 g body weight) were purchased from Charles River Laboratories (Wilmington, MA, USA). Rats were housed in standard 12-hour cycle lighting with food and water provided *ad libitum* prior to and throughout the experimental protocol. Surgery was performed under isoflurane inhalation anesthesia (100% O_2_, 5% isoflurane for induction, 2% for maintenance), and all efforts were made to minimize suffering.

### AAV Vector Production

Recombinant AAV vectors used in this study were prepared in our laboratory by helper-free triple-plasmid transfection of 293T cells [30], followed by Optiprep (Sigma-Aldrich, St Louis, MO, USA) gradient purification according to a protocol as described previously [31], with some minor modifications. The plasmids used for AAV production include: 1) pAAV-CMV-EGFP expression plasmid contains the self-complementary (sc) AAV2 inverted terminal repeats (ITR) and codes EGFP downstream of a chimeric intron enhancing transcription driven by CMV promoter [32]; 2) pRep2/Cap6 (pRC6) and pRC8 (Viromics, Fremont, CA, USA) containing AAV2 replication (*rep)* and capsid (*cap)* protein genes from either AAV6 or AAV8, respectively; and 3) pHelper (Viromics) encoding the adenoviral helper genes. AAV vectors were produced by CaPO4 cotransfection of pAAV-CMV-EGFP with either pRC6 or pRC8, and pHelper plasmids into the 293T cells. Three days after transfection, the cell pellets were resuspended in the lysis buffer (50 mM Tris-HCl,150 mM NaCl, 2 mM MgCl_2_, pH 8.0), and lysed by 3-cycles of freeze-thaw. Cell lysates clarified by centrifugation at 2500 *g* for 30 min were incubated with 100 U/ml of benzonase (Sigma-Aldrich) for 1 h at 37°C. A stock solution of 40% PEG 8000 (Sigma- Aldrich) containing 2.5 N NaCl was added to the clarified cell lysate to final concentration of 8% [33]. The solution was then incubated on ice for 2 h, centrifuged at 2500 *g* for 30 min, and the pellet was resolved in phosphate-buffered saline (PBS) containing 1 mM CaCl_2_ and 2 mM KCl. The AAV particles were purified with an Optiprep-based process. As detailed below, Optiprep discontinuous gradients were formed in 40 ml of Quick-Seal centrifuge tubes (Beckman Instruments, Palo Alto, CA, USA). The steps of the gradient were 10 ml of 15%, 8 ml of 25%, 6 ml of 40%, and 2 ml of 60% Optiprep. Fifteen milliliters of the AAV virus lysate was then overlaid onto the gradient. The sealed tubes were centrifuged for 70 min at 350,000 *g* in a 70 Ti rotor (Beckman Instruments) at 18°C and the virus was extracted with an 18-gauge needle from the 40–60% interphase and lower ½ portion of the 40% layer. The obtained viral suspension was concentrated with Centricon Plus-20 (Regenerated Cellulose 100,000 MWCO, Millipore, Billerica, MA), and the final viral preparations were kept in PBS containing 5% sorbitol (Sigma-Aldrich) and stored in −80°C. Encapsidated DNA (vector genome) was determined relative to plasmid standards by a PicoGreen assay (Invitrogen, Carlsbad, CA, USA) and also dot-blot following denaturation of the AAV particles [34], [35]. AAV plasmid contamination in the encapsidated vector genome of purified AAVs was not detectable by PCR using a primer pair for the ampicillin resistance gene. The physical particle titers were calculated and expressed as genome copy number per ml (GC/ml). The titers of AAV6-EGFP and AAV8-EGFP vectors were 2.1×10^13^ GC/ml and 2.5×10^13^ GC/ml, respectively. The same lot of viral preparation was used for all *in vivo* experiment.

### Integrity of Purified Vectors: Purity and Full Particle Content

Vectors were evaluated for purity by sodium dodecyl sulfate–polyacrylamide gel (SDS-PAGE) electrophoresis followed by silver stain using a Pierce silver stain kit (Fisher Scientific, Rockford, IL) according to manufacturer’s protocol. In addition, viral capsid proteins were confirmed by standard immunoblot procedures using a mouse anti-Vp1, -2, -3 antibody (1∶1000 dilution; Research Diagnostic Inc.). The ratios of Vp1:Vp2:Vp3 proteins and the purity relative to nonvector proteins visible on stained gels were quantified with NIH ImageJ software (http://rsbweb.nih.gov/ij/). Negative staining and electron microscopy (EM) scan were performed to assess particle content of purified AAV vectors. Copper grids (400-mesh coated with a formvar–thin carbon film; Electron Microscopy Sciences, Hatfield, PA) were pretreated with 1% Alcian blue (Electron Microscopy Sciences) and loaded with 5 **µ**l of vector preparation. The grids were then washed, stained with 1% uranyl acetate (Electron Microscopy Sciences), and viewed with a JEOL 2100 & Hitachi 600 transmission electron microscope. Empty-to-full particle ratios were determined by direct counting of the electron micrographs.

### Microinjection of Viral Vectors into the DRG

The AAV vectors of AAV6-EGFP or AAV8-EGFP were microinjected unilaterally into the lumbar (L) 4 and L5 DRGs using our previously described techniques [2]. Briefly, after exposure, the intervertebral foramen was enlarged by removal of just enough laminar bone to expose the distal pole of the DRG. A pulled glass micropipette was advanced through the capsule and approximately 100 µm into the ganglion. Injection of vehicle (5% sorbitol in PBS) and AAV vectors at the adjusted titer of 2×10^9^ viral particle in 2 µl per DRG was performed over a 5 min period using a Nanoliter 2000 microprocessor-controlled injector (World Precision Instruments, Sarasota, FL, USA). This volume has been demonstrated to fill the DRG without spilling into adjacent tissues [2]. Removal of the pipette was delayed for 5 min to minimize reflux. Following the injection, the animals were returned to the animal house until the time of transcardial perfusion, fixation, and collection of fixed tissues for immunohistochemical analysis.

### Histology and Immunohistochemistry (IHC)

All histology and IHC staining were performed by a standard previously described protocol [36]. Briefly, the ipsilateral and contralateral DRGs, the corresponding levels of the spinal cord, the sciatic nerve at middle thigh level, and the medial plantar skin of hindpaws (L4–L5 dermatomes) were removed and fixed in Zinc-formalin, embedded with paraffin, and prepared into a series of 5 µm sections. Sections were de-waxed, and antigen retrieval was achieved by microwave heating in citrate buffer. Sections were then immunolabeled with the antibodies against GFP, β3-tubulin, calcitonin gene related peptide (CGRP), tyrosine kinase receptor type 1 (TrkA), neurofilament 200 (NF200), Ca^2+^/Calmodulin-dependent kinase II (CaMKII), neuronal nuclei (NeuN), glial fibrillary acidic protein (GFAP), glutamine synthetase (GS), Ionized calcium binding adaptor molecule-1 (Iba1), activating transcription factor 3 (ATF3), CD6 and CD8, and biotinylated-isolectin-B4 (IB4) binding, with BSA replacement of first antibody as the negative control. The appropriate fluorophore-conjugated secondary antibodies were used to reveal the primary antibodies. (Antibody catalogue, immunogen and dilution details are presented in **Table 1**). The sections were examined and images captured under a Nikon TE2000-S fluorescence microscope with filters suitable for selectively detecting the green and red fluorescence using an Optronics QuantiFire digital camera, or were examined under a light microscope. For double label co-localization, images from the same section but showing different antigen signals were overlaid. Apoptosis in DRG sections was assessed with the terminal deoxynucleotidyl transferase-mediated dUTP nick-end labeling (TUNEL) method using the *in situ* cell death detection kit (Roche, Indianapolis, IN) according to the instructions of the manufacturer.

**Table 1:** Antibodies used in immunohistochemistry.

Primary antibody^1^	Cat#	Target	Dilution	Vendor^2^	Second antibody^3^
Ms anti-GFP	sc-9996	GFP	1∶400	SCB	Dk anti-Ms IgG conjugated with 488 (1∶2000)
Rb anti-GFP	2555	GFP	1∶400	CS	Dk anti-Rb IgG conjugated with 488 (1∶2000)
Ms anti-β3-tubulin	sc-80016	All neurons	1∶500	SCB	Dk anti-Ms IgG conjugated with 549 (1∶2000)
Ms anti-NF200	N0142	A-neurons	1∶1000	Sigma	Dk anti-Ms IgG conjugated with 549 (1∶2000)
Ms anti-CGRP	sc-57053	Nociceotiveneurons	1∶600	SCB	Dk anti-Ms IgG conjugated with 549 (1∶2000)
Biotinylated-IB4	I21414	Nociceptiveneurons	10 µg/ml	Invitrogen	SP conjugated with 549 (1∶6000)
Rb anti-TrkA	sc-118	Nociceptiveneurons	1∶400	SCB	Dk anti-Rb IgG conjugated with Cy3 (1∶2000)
Ms anti-NeuN	MAB337	All neurons	1∶100	Millipore	Dk anti-Ms IgG conjugated with 549 (1∶2000)
Rb anti-CaMKII	sc-176	Neurons	1∶500	SCB	Dk anti-Rb IgG conjugated with Cy3 (1∶2000)
Rb anti-Iba1	019-19741	Microglia	1∶4000	Dako	Dk anti-Rb IgG conjugated with Cy3 (1∶2000)
Rb anti-GS	sc-9067	Satellite glia	1∶600	SCB	Dk anti-Rb IgG conjugated with 549 (1∶2000)
Rb anti-GFAP	2015-02	Satellite glia	1∶4000	Dako	Dk anti-Rb IgG conjugated with Cy3 (1∶2000)
Ms anti-CD6	CBL554	Pan T-cells	1∶100	Millipore	Dk anti-Ms IgG conjugated with 549 (1∶2000)
Ms anti-CD8	217580	Cytotoxic T-cells	1∶100	Millipore	Dk anti-Ms IgG conjugated with 549 (1∶2000)

1 Ms, mouse; Rb, rabbit.

2 SCB: Santa Cruz Biotechnology, Santa Cruz, CA; Sigma: Sigma-Aldrich, St. Louis, MO; CS: Cell signaling, Danvers, MA; Invitrogen: Invitrogen, Carlsbad, CA; Millipore: Billerica, MA; Wako: Wako Chemicals USA, Richmond, VA; Dako: Carpinteria, California.

3 Dk, Donkey. All secondary antibodies are from Jackson Immunoresearch, West Grove, PA.

### Measurement and Quantification of Immunostaining

EGFP-positive neurons were defined as cells with the fluorescence intensity greater than average background fluorescence plus 2 standard deviations of neurons in a section from a naïve animal (no injections) under identical acquisition parameters (n = 250, identified by β3-tubulin staining at a different wavelength), a method adapted from Vulchanova, *et al* [13]. DRGs (both L4 and L5) from four animals selected randomly were analyzed for histological quantification as described previously [36]. For quantification of transduction efficiency, every tenth DRG section was selected from the consecutive serial sections (3 to 5 sections for each DRG), and in each selected section, the number of EGFP labeled cells was counted and transduction efficiency was expressed as the percentage of total neuronal profiles revealed by β3-tubulin staining. Every section was photographed at fixed exposure settings and 10×magnification, which encompassed more than 200 neuron profiles in each section. To construct profile size distribution histograms, the cross-sectional area of EGFP-labeled neurons for which nuclei were evident was measured using Adobe Photoshop CS3 (Adobe Systems Incorporated). Neurons were divided into three size groups: small (<300 µm^2^), medium (300–700 µm^2^) and large (>700 µm^2^) neurons as described previously [37], [38]. To determine the proportion of a given marker population expressing GFP, positively immunostained neuronal profiles for each marker were identified and the overlaid images were used to count double-labeled neurons.

For quantitative analysis of GS and GFAP in DRG sections, neurons with a visible nucleus and surrounding satellite cells were photographed under 40×magnification, manually selected using Trace region command in a Metamorph software 7.1.4. (Molecular Devices, Downington, PA). The area of fluorescence for specified regions of interest was quantified using automatic threshold settings that were kept constant across images. The main output measure of GS and GFAP staining was the ratio of satellite cell area divided by the area of the individual neuron that they surrounded. For Iba1, the measure was total immunopositive area per field. Quantification of the percentage of ATF3-induced DRG neurons was determined by counting the number of ATF3 immunoreactive and non-immunoreactive neurons with visible nuclei marked by Hoechst and EGFP staining (in EGFP negative sections, low level of background EGFP staining enables negative neurons and their nuclei to be clearly visualized). The percentage of ATF3 neurons out of total neurons and the percentage of ATF3-inducted neurons expressing EGFP were counted by switching between Hoechst, 488, and 549 filter images on randomly chosen sections from L5. For quantification GFAP, GS and Iba1 in spinal cord sections, the fluorescence intensity values were acquired along a line positioned in each section between the dorsal root entry zone and the central canal (Line scan function, scan width 50 pixels). The average intensity across the full length of this line was then determined for each section. Only sections with EGFP fluorescence were used for measurement. For control spinal cords, only one arbitrarily chosen side was measured.

### Behavioral Evaluation

Behavioral tests were carried out before (baseline) and after vehicle or AAV vector injection at the indicated days thereafter, as previously described [2]. Mechanical withdrawal threshold testing (von Frey) was performed using calibrated monofilaments (Patterson Medical, Bolingbrook, Illinois, USA). Briefly, beginning with the 2.8 g filament, filaments were applied with just enough force to bend the fiber and held for 1 s. If a response was observed, the next weaker filament was applied, and if no response was observed, the next stiffer filament was applied, until a reversal occurred, defined as a withdrawal after a previous lack of withdrawal, or *vice versa*. Following a reversal event, four more stimulations were performed following the same pattern. The forces of the filaments before and after the reversal, and the four filaments applied following the reversal, were used to calculate the 50% withdrawal threshold according to the method of Dixon [39]. Rats not responding to any filament were assigned a score of 25 g. Noxious punctate mechanical stimulation (pin test) was performed using the point of a 22 g spinal anesthesia needle, which was applied to the center of the hindpaw with enough force to indent the skin but not puncture it. This was applied for 5 applications separated by at least 10 s, which was repeated after 2 min, making a total of 10 touches. For each application, the induced behavior was either a very brisk simple withdrawal with immediate return of the foot to the cage floor, or a sustained elevation that included licking and chewing, and possibly shaking, which lasted at least 1 s, characteristic of hyperalgesic behavior [40], [41]. The degree of hyperalgesia was recorded as the percentage of total touches.

### Data Analysis

Data are expressed as means ± SD. A probability of p<0.05 was considered significant. The statistical comparison of *in vivo* transduction rates between AAV6-EGFP and AAV8-EGFP was analyzed by 2-way ANOVA (for vector and time after injection) with *post hoc* paired comparisons performed by Tukey’s test where appropriate. Expression of GS, GFAP, and Iba1 in different comparative groups were analyzed by Kruskal-Wallis test, with *post hoc* paired comparisons performed by Dunn’ test where appropriate. Expression of ATF3 in different DRG groups was analyzed using Chi Square for main effect, and paired comparisons using Fisher’s Exact test corrected by Bonferroni’s method. The Kolmogorov-Smirnov (KS) test was used for statistical analysis of the distribution of cell size frequency between EGFP-positive neuronal and total neuronal populations. Behavioral changes over time in each group were analyzed by repeated measures parametric ANOVA for von Frey testing with *post hoc* paired comparison by Tukey’s test, and non-parametric Friedman’s ANOVA for pin testing with *post hoc* paired comparison by Wilcoxon signed-rank test. To compare between vectors, the time course data were also analyzed using area under the curve (AUC) across the 4-week testing period for measures normalized to day 0, with differences between groups tested by Mann-Whitney paired comparisons corrected by Bonferroni’s technique. All statistical analysis was performed using Statistica (StatSoft, Tulsa, OK, USA).

## Results

### Assessment of Integrity of Vector Preparation

**Figure 1:**
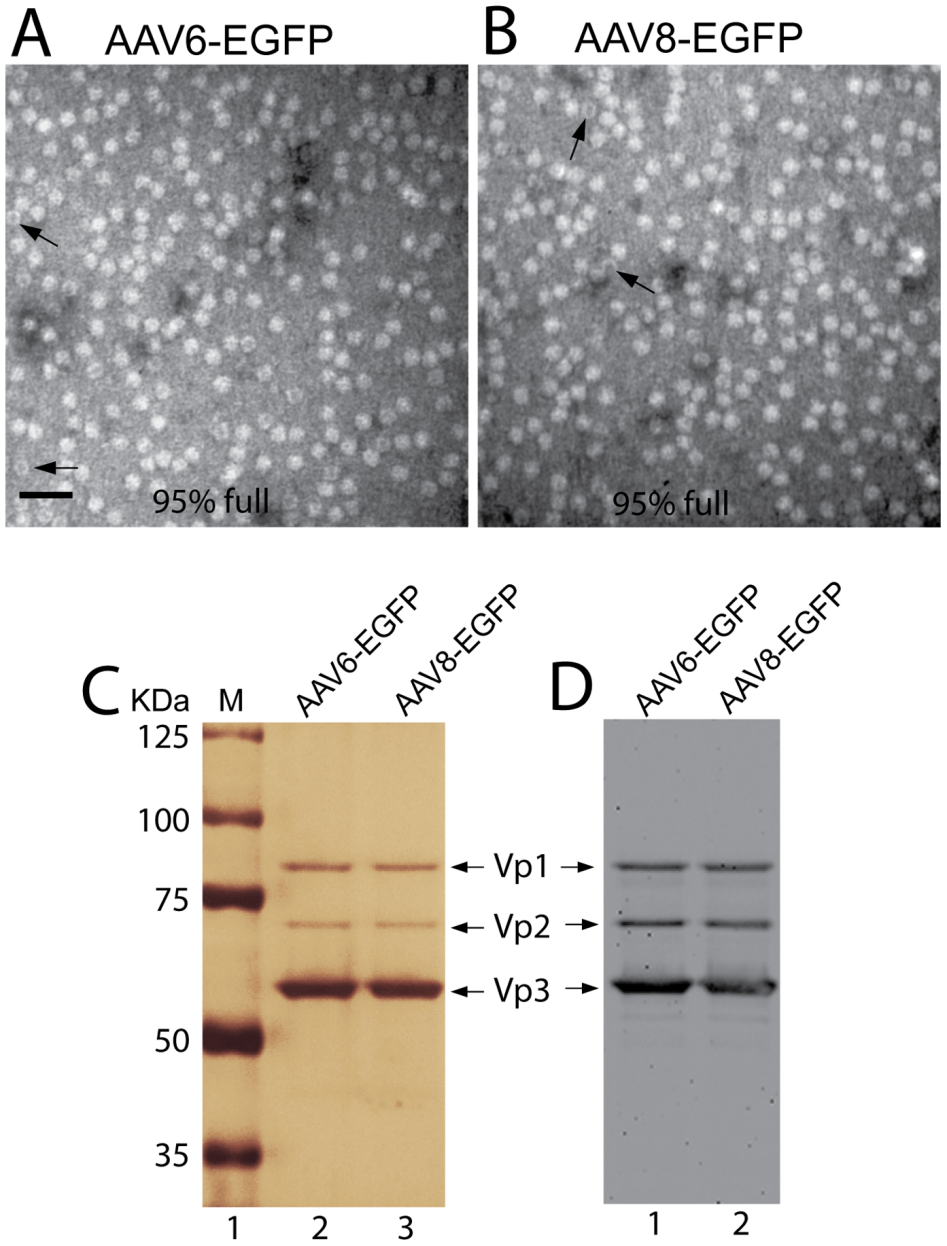
Integrity of purified AAV6-EGFP and AAV8-EGFP vectors. Purified AAV6-EGFP (**A**) and AAV8-EGFP (**B**) vectors were negatively stained with uranyl acetate and examined by transmission electron microscopy. Empty particles (arrows) can be distinguished on the basis of the electron dense center and are indicated by arrows. Properly DNA-filled particles made up 95% of the total particles in each case. Silver stain (**C)** was performed on SDS-PAGE after loading equally with 5×10^9^ genome-containing particles of purified AAV6-EGFP and AAV8-EGFP. This revealed 3 virion protein bands of Vp1, Vp2, and Vp3, with molecular weight of 87, 72 and 62 KDa, respectively, in each sample, corresponding to the particular AAV capsid proteins revealed by immunoblot using AAV specific antibody (**D**). The absence of other protein bands demonstrates the high purity of the preparations.

The integrity of purified AAV vectors, including the purity and empty-to-full particle ratios, has important implications for both safety and efficacy of *in vivo* gene transfer [27], [28], [29]. In order to compare the efficacy and safety of AAV6-EGFP and AAV8-EGFP accurately, we initially aimed to ensure that the purified vectors were similar in quality so that differences between the two vectors would be solely attributable to the particle capsids rather than other differences in the preparations. The Optiprep discontinuous gradient ultracentrifugation protocol that we used for vector purification has been shown to separate empty from full viral particles [42], [43]. The resultant titers of AAV6-EGFP and AAV8-EGFP were 2.1×10^13^ GC/ml and 2.5×10^13^ GC/ml, respectively, with less than 5% of particles being empty (i.e. virus protein shells without the vector genome) for both vectors (**Fig. 1A and 1B**). Silver staining of SDS-PAGE gel loaded with equal 5×10^9^ viral particles for purified AAV6-EGFP and AAV8-EGFP revealed 1∶1:10 ratios of Vp1, Vp2, and Vp3 of AAV virion capsid proteins, which comprised >95% of total silver-stained proteins, for both vectors (**Fig. 1C and 1D**). These results indicate that one-step Optiprep gradient is sufficient to yield high-purity and high-titer AAV particles without the necessity of further chromatographic purification [**44**], and that the viral integrity (i.e. purity and the ratio of properly DNA-filled particles) for AAV6-EGFP and AAV8-EGFP vectors used in this study were comparable.

### Transduction of DRG Neurons by AAV6-EGFP and AAV8-EGFP

**Figure 2:**
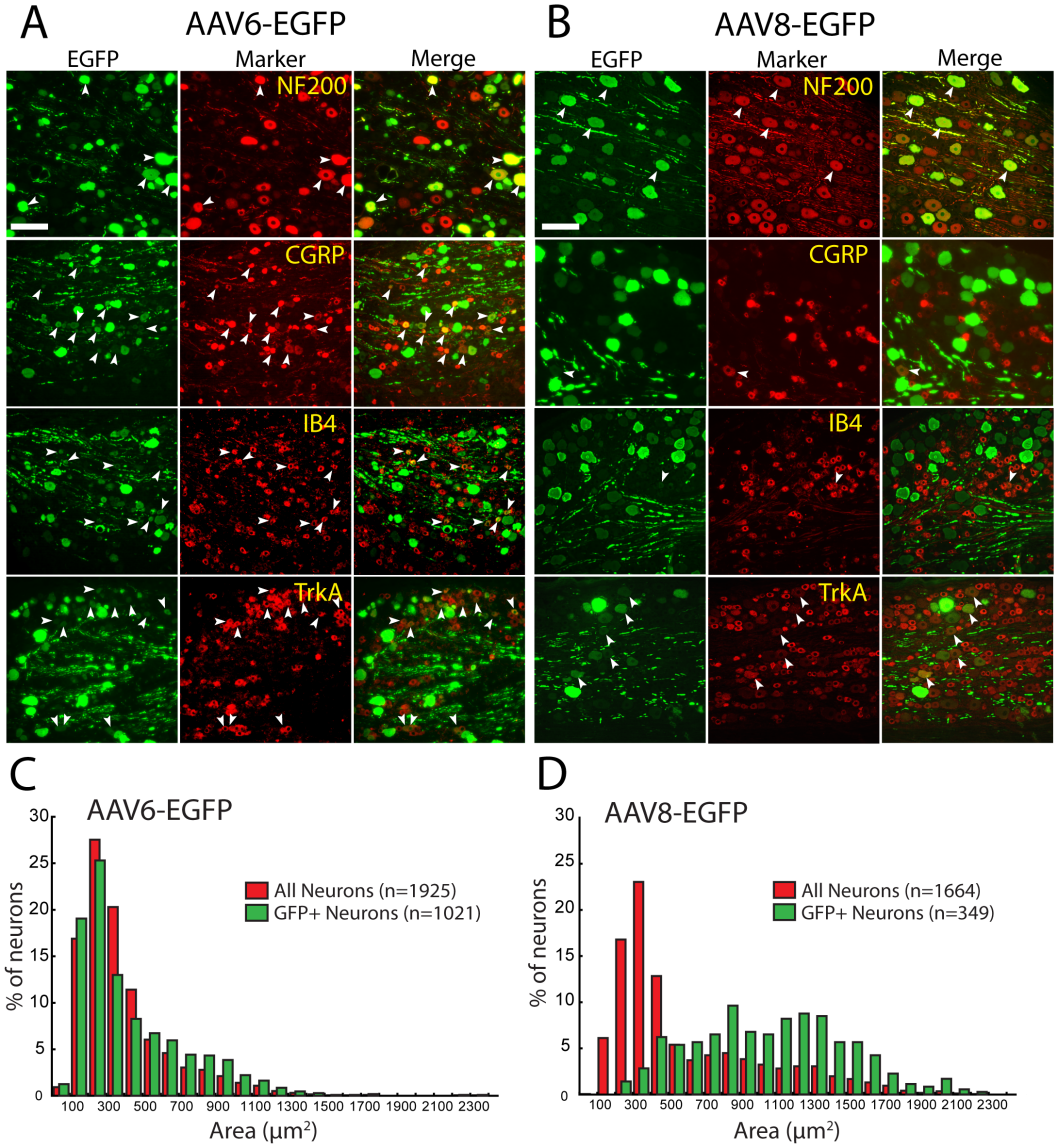
AAV6-EGFP and AAV8-EGFP transduction in DRG. DRG sections 3 weeks after AAV6-EGFP injection (**A**) or AAV8-EGFP injection (**B**) were immunostained with antibodies to GFP and the indicated markers. Co-localization is shown in the merged images. Arrowheads point to the same neuron when stained for EGFP, the specified marker, and in the merged image. Scale bars: 100 µm for all images. DRG neuronal size histograms 3-weeks following DRG injection of AAV6-EGFP (**C**) and AAV8-EGFP (**D**) show the distribution frequencies for transduced somata (green bars) compared to the total DRG neuron population identified by β3-tubulin staining (red bars).

To compare *in vivo* transduction efficiency and tropism of these vectors in the peripheral sensory system of adult rats, purified AAV6-EGFP or AAV8-EGFP vector was injected into the L4 and L5 DRGs (same vector in both L4 and L5 of any animal) using a well-characterized technique [2]. EGFP expression was first evident in the DRG at 1 week following vector administration for both vectors, although the intensity of EGFP immunoreactivity at this time point was variable (not shown). A robust EGFP expression for both vectors was observed at 2 to 4 weeks after injection. EGFP expression was restricted to the DRG neuronal somata and projections, and no EGFP could be identified in satellite glia or other non-neuronal cells (**Fig. 2A, B**; also see below). The size of neurons transduced by AAV6-EGFP (n = 1021 neurons) and AAV8-EGFP (n = 349) were compared to the total DRG neuronal populations identified by neuron-specific β3-tubulin immunostaining. The frequency distribution of cell size for neurons transduced by AAV6-EGFP (**Fig. 2C**) was comparable to the size distribution for all neurons (p>0.05 by KS test between EGFP-positive neuronal population *versus* total neuronal population), showing approximately 80% of small- and medium-sized neurons and approximately 20% of large-sized neurons for both total neurons and EGFP-positive neurons. Double immunofluorescence examination (**Fig. 2A**) revealed that 44±9% (308/717) of neurons expressing EGFP co-labeled with the peptidergic nonmyelinated neuron marker CGRP, 23±8% (127/532) with the non-peptidergic nonmyelinated marker IB4, and 60±17% (144/246) with nociceptive maker TrkA. Also, 28±7% (220/811) of transduced neurons co-labeled with NF200, a marker of myelinated A-fiber neurons including proprioceptive neurons [17]. In these specific subpopulations, rates of transduction by AAV6-EGFP were 53±15% (308/601) of CGRP-positive neurons, 29±17% (127/428) of IB4-positive neurons, 36±10% (144/434) of TrkA-positive neurons, and 62±12% (220/357) of NF200-positive neurons.

In contrast to the AAV6-EGFP transduction profile, AAV8-EGFP disproportionately transduced large neurons (**Fig. 2D**) (p<0.01 by KS test between EGFP-positive neuronal population *versus* total neuronal population). Double immunofluorescence examination (**Fig. 2B**) revealed that 91±8% (210/235) of the EGFP-positive neurons were co-labeled with NF200, but few neurons co-labeled with nociceptor markers of CGRP (5±4%, 18/379) or IB4 (5±3%, 16/340), while 39±10% (50/155) of EGFP-positive neurons co-labeled with TrkA, typically medium/large-sized neurons. In specific neuronal subpopulation, rates of transduction by AAV8-EGFP were 2±1% (18/763) of CGRP-positive neurons, 3±2% (16/707) of IB4-positive neurons, 10±7% (50/571) of TrkA-positive neurons, and 52±17% (210/441) of NF200-positive neurons. These results indicate that intraganglionic AAV6 efficiently transduces the global neuronal population while AAV8, although strongly selective for neurons after DRG injection, shows preferential neuronal tropism for large-sized myelinated sensory neurons.

**Figure 3:**
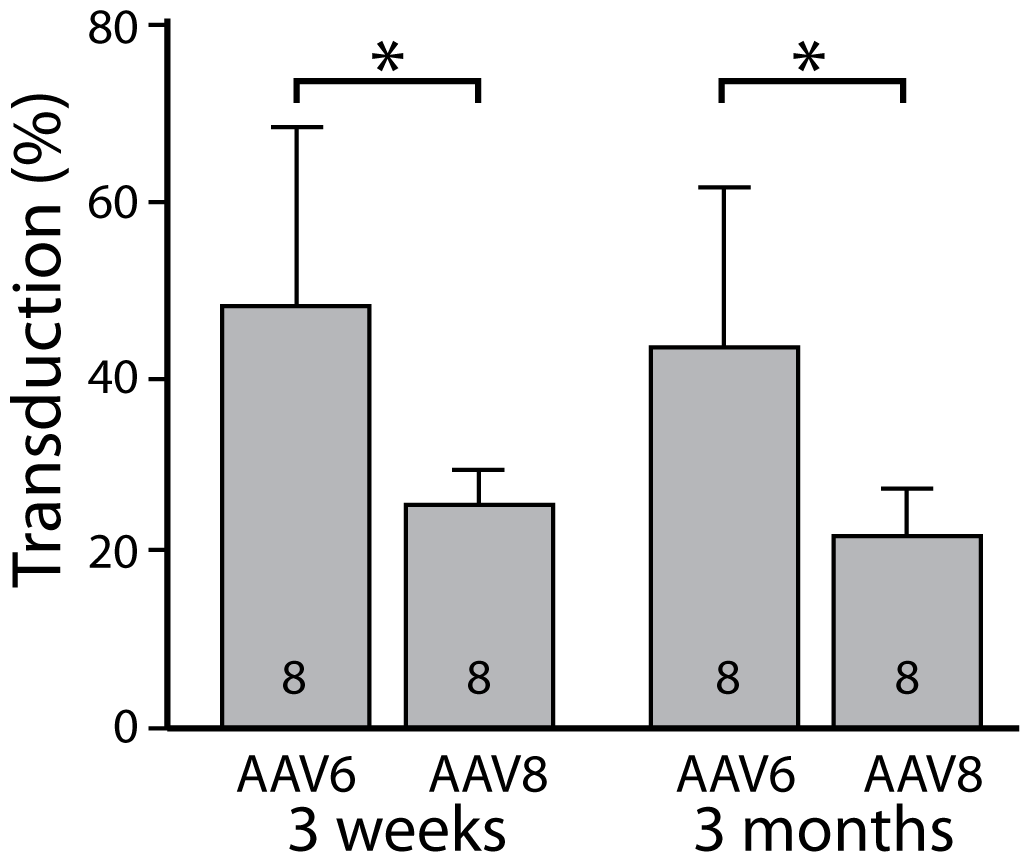
Sustained EGFP expression. Bar graphs represent the percentage of *in vivo* transduction rates per total DRG neurons of AAV6-EGFP and AAV8-EGFP 3 weeks (n = 8 DRGs from 4 rats per vector) and 3 months (n = 8 DRGs from 4 rats per vector) after injection. Two-way ANOVA identified a main effect of vector (p<0.001) and significant paired comparisons at each time point (*, p<0.05). Data are presented as mean ± SD.

Both L4 and L5 DRGs from 4 rats at 3 weeks and 4 rats at 3 months (the latest time point) after injection with AAV6-EGFP and AAV8-EGFP were analyzed by histological quantification for *in vivo* transduction efficiency. Comparison of transduction rates for AAV6-EGFP *versus* AAV8-EGFP (**Fig. 3**) showed a main effect of vector (p<0.001), with significant paired comparisons at 3 weeks (p<0.05) and 3 months (p<0.05) at which AAV6-EGFP produced higher transduction rates than the corresponding values for AAV8-EGFP. No significant reduction of transduction rate at 3-month was noted, compared to 3-week, for either AAV6-EGFP or AAV6-EGFP. Results indicate that both AAV6 and AAV8 mediated stable and prolonged *in vivo* transfer of EGFP to DRG neurons, and that transduction rates of AAV6 exceed that of AAV8.

### EGFP is Expressed in Central and Peripheral Processes of Transduced Sensory Neurons

**Figure 4:**
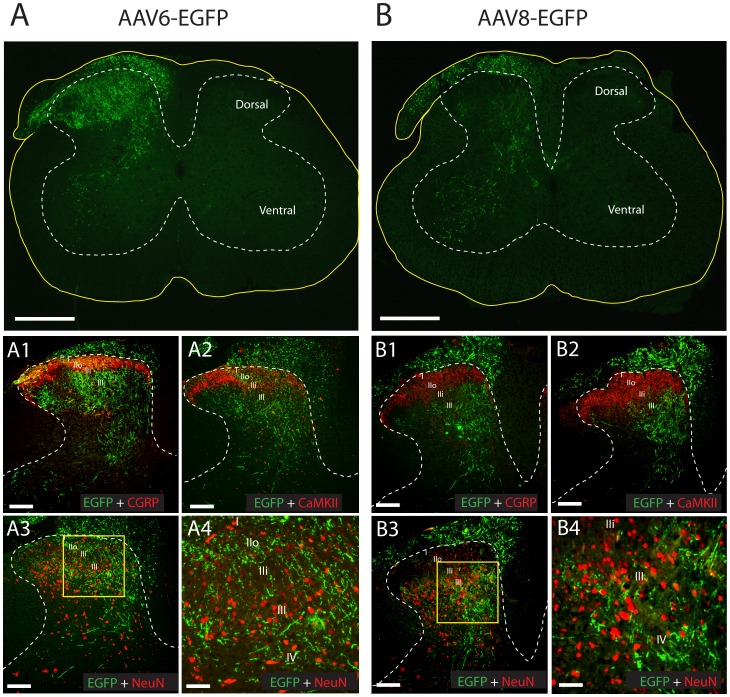
Central termination patterns of EGFP-positive axons in the spinal cord. Representative low-power views of transverse spinal cord sections at fourth and fifth lumbar levels show EGFP (green) expression 3 weeks following injection of AAV6-EGFP (**A**) and AAV8-EGFP (**B**). Yellow lines indicate the spinal cord transverse section profiles and white dashed lines indicate cord grey matter. Additional images show co-labeling of EGFP (green), CGRP (red), CaMKII (red), and NeuN (red) in the dorsal horn for AAV6-EGFP (**A1, A2**, and **A3**) and AAV8-EGFP (**B1, B2** and **B3**). White dashed lines indicate dorsal horns, with laminae I-III marked in each section. The boxed areas in **A3** and **B3** are shown at higher magnification, which demonstrate that vectors AAV6-EGFP (**A4**) and AAV8-EGFP (**B4**) transduced no dorsal horn neuronal somata immunolabeled by NeuN. Scale bars: **A** and **B**, 500 µm; **A1**, **A2**, **A3**, **B1**, **B2**, and **B3**, 100 µm; **A4** and **B4**, 50 µm.

Immunohistochemistry of the lumbar spinal cord sections revealed EGFP fluorescence-highlighting fibers of DRG neuron projections entering the spinal cord. The populations of neurons transduced by AAV6-EGFP and AAV8-EGFP exhibited distinct patterns of distribution. At L4 and L5 spinal cord levels, EGFP-expressing fibers were observed in all dorsal horn laminae ipsilateral to the intraganglionic injection of AAV6-EGFP (**Fig. 4A**), but were particularly abundant in the superficial dorsal horn (lamina I and outer lamina II) identified by CGRP immunolabeling (**Fig. 4A** **1**). Using CaMKII, an alternative marker of these laminae, confirmed projections of AAV6-transduced neurons to the superficial dorsal horn (**Fig. 4A** **2**). Fibers from AAV6-transduced neurons were also evident in ipsilateral dorsal columns, deeper laminae III-V (nucleus proprius), and the ventral horn, as well as in the attached nerve roots (**Fig. 4A**). No dorsal horn neurons, labeled with NeuN, showed evidence of EGFP expression (**Fig. 4A** **3, 4**). In contrast to AAV6-EGFP, AAV8-EGFP transduced few afferents that projected to the superficial laminae of the dorsal horn (**Fig. 4B** **1** and **4B2**). Instead, EGFP immunoreactivity in the dorsal horn was mostly present in the deeper laminae (III-V), as well as significant projections extending into the ventral horn (**Fig. 4B**). Like AAV6-EGFP, AAV8-EGFP transduced no dorsal horn neuronal somata immunolabeled by NeuN (**Fig. 4B** **3, 4**), which indicates that the EGFP transgene product reached presynaptic sites but was not transported across central synapses. For both vectors, few EGFP-positive fibers were detectable at thoracic and sacral levels in the dorsal column, but no EGFP signals were observed in the contralateral spinal cord sections at these levels (data not shown), suggesting that vector-mediated EGFP transduction in the central terminals was limited to the ipsilateral lumbar segment of the spinal cord.

**Figure 5:**
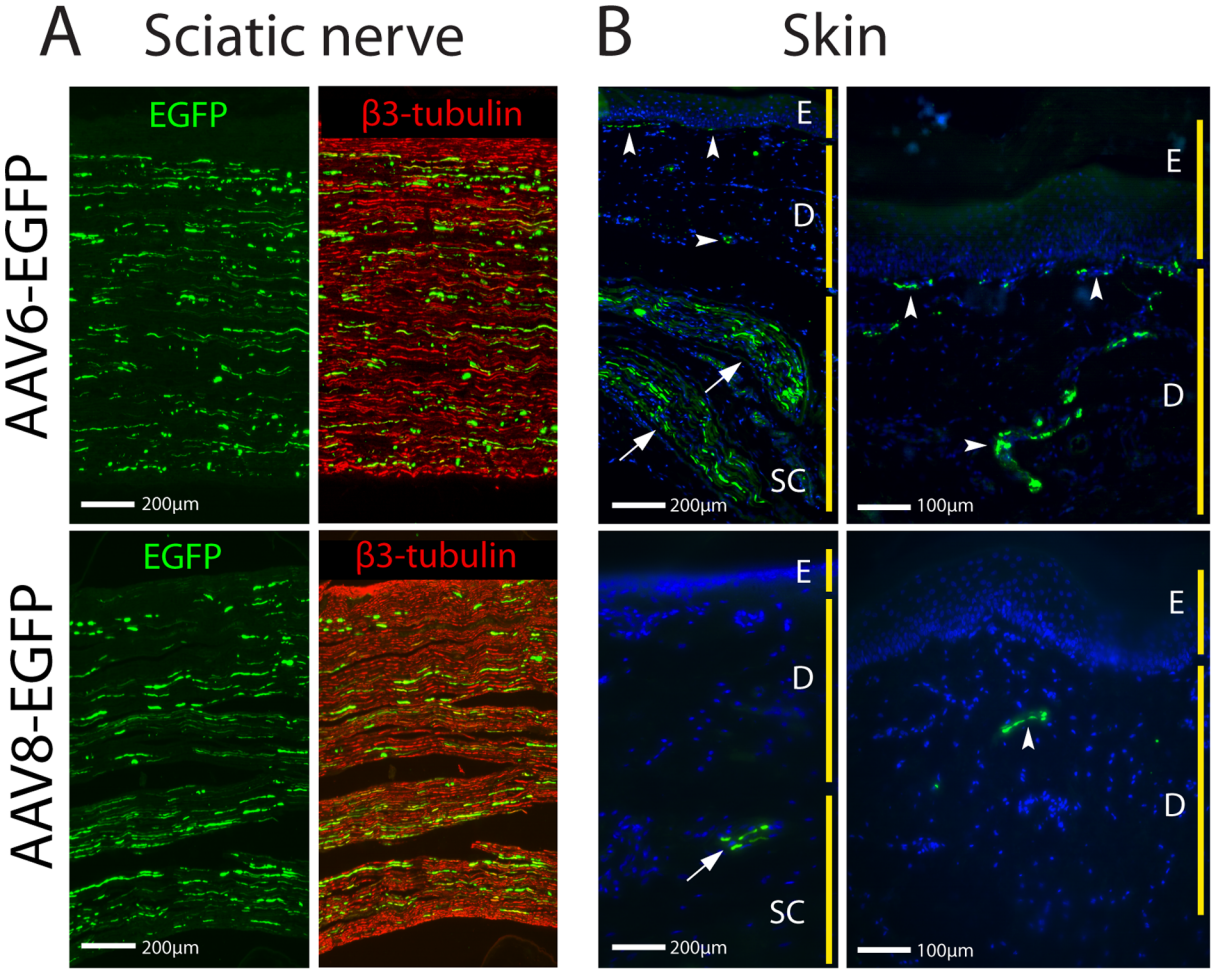
EGFP expression in sciatic nerve and cutaneous tissue. Longitudinal sciatic nerve sections (**A**) reveal immunofluorescence for EGFP expression (green) in a subpopulation of β3-tubulin-positive fibers (red) 3 weeks after intraganglionic injection of AAV6-EGFP (top) and AAV8-EGFP (bottom). In the medial plantar skin (**B**, low power on left, higher power on right), AAV6-EGFP sections (top) show numerous thick bundles of EGFP-positive nerve fibers (green; Hoechst counterstain blue) in the subcutaneous layer (arrows), and EGFP-positive thinner nerve fibers were also observed in the dermis and dermoepidermal junction (arrowheads). However, AAV8-EGFP sections (bottom) show only few EGFP-positive fibers observed in the subcutaneous layer (arrow) and dermis (arrowhead). Labels: E, epidermis; D, dermis; and SC, subcutaneous layer.

Sciatic nerve and medial plantar cutaneous tissues of hindpaws corresponding L4–L5 dermatomes were sectioned to examine EGFP spread along peripheral axonal processes. Intense EGFP immunofluorescence was found in sections of the ipsilateral sciatic nerve at middle thigh level following DRG injection of either AAV6-EGFP or AAV8-EGFP (**Fig. 5A** **,** n = 3 for each vector). In the medial plantar skin (**Fig. 5B** **,** n = 3 for each vector), numerous thick bundles of EGFP-positive nerve fibers present in the subcutaneous layer and EGFP-positive thinner nerve fascicles were also observed in the dermis and dermoepidermal junction from AAV6-EGFP, but few EGFP-positive nerve fascicles were seen in AAV8-EGFP injected rats.

### Lack of Apparent Inflammatory, Cellular Immunity, or Neurotoxicity Response

**Figure 6:**
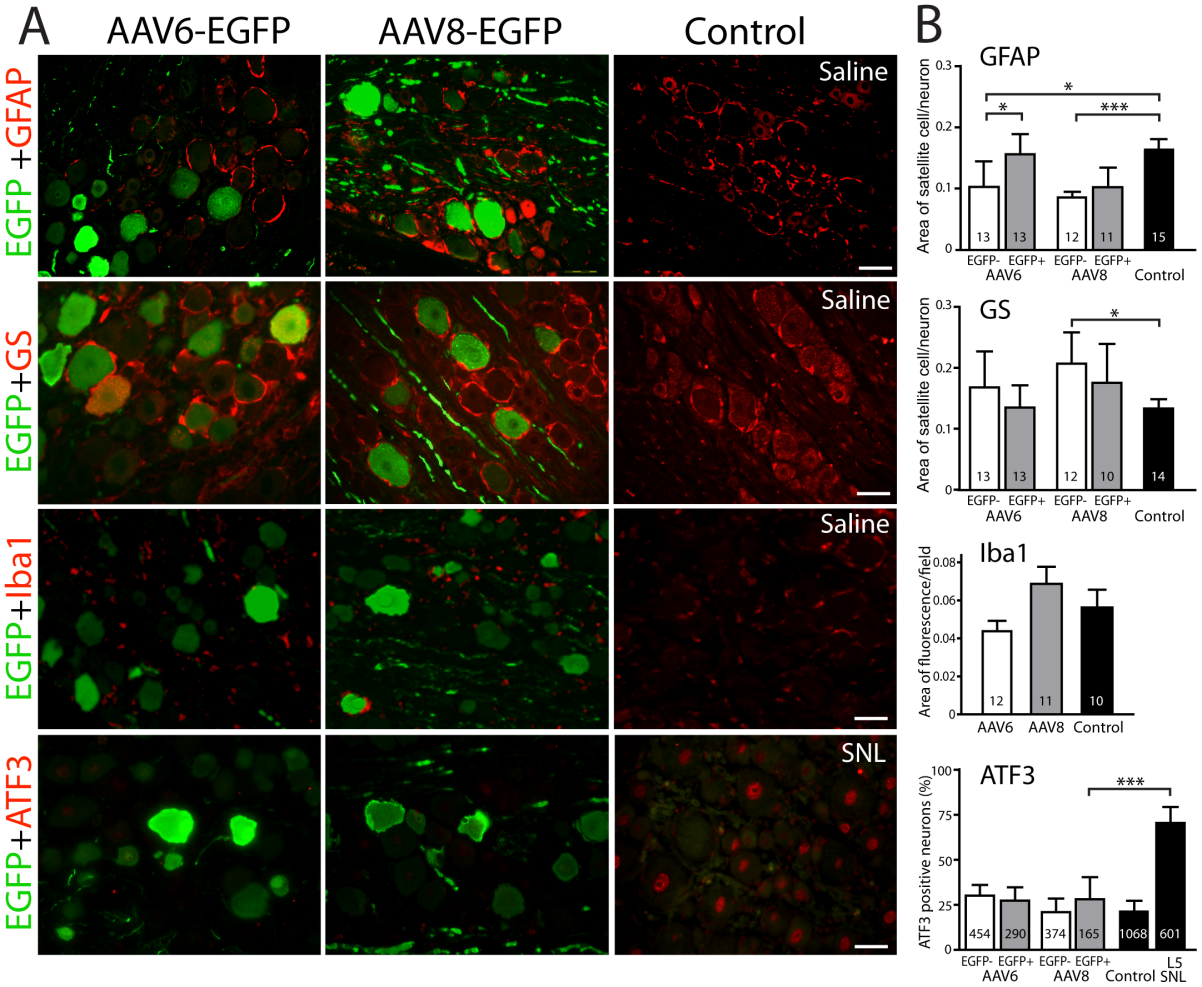
Identification of inflammatory response and neurotoxicity in AAV injected DRGs. **A.** Representative examples of GFAP (top row), GS (second row), Iba1 (third row), and ATF3 (bottom row) immunoreactivity in dorsal root ganglia 4 weeks after injection of AAV6-EGFP (first column), AAV8-EGFP (second column), and control condition (third column), which is saline injection for GFAP, GS, and Iba1, but spinal nerve ligation (SNL) as a positive control for ATF3. Samples are co-stained for EGFP immunoreactivity to identify transduced neurons. AAV-injected DRG neurons exhibit significantly less ATF3-positive nuclei in comparison to the SNL group. Scale bar: 50 µm. **B.** Quantification of immunoreactivity for each marker. For GFAP and GS, the measurement is the ratio of satellite glial cell area (identified by GFAP or GS) divided by the area of the neuron they surround. For Iba1, the area of immunopositive microglia (identified by Iba1) as a fraction of the total field is recorded. For ATF3, the percent of positive cells is recorded. The number in each column is the number neurons for each group for GFAP, GS, and ATF3, and fields for Iba1, which were derived from 4–7 different sections and at least 4 animals per group. Data are presented as mean ± SD. *p<0.05, ***p<0.001.

L5 DRGs and spinal cords at 4-week post-injection (n = 3 for each vector) were examined for evidence whether inflammation, cellular immune reaction, and neurotoxicity had been triggered by vector injection. In DRGs, hematoxylin & eosin staining indicated that neurons retained their structural integrity and leukocytes were absent after injection of either vector, and TUNEL staining revealed no apoptosis in neurons or satellite glial cells following injections (data not shown). For IHC examination of DRGs, negative controls consisted of saline-injected DRGs, while positive control sections obtained following spinal nerve ligation (L5 SNL) provided assurance of the adequacy of the staining technique and antibodies. Vector injection produced images that differed minimally from the negative control for the various markers of inflammation, immune activation, and neurotoxicity (**Fig. 6A**). Quantification (**Fig. 6B**) revealed that the area of GFAP-positive satellite glial cell rings was not increased by injection of either vector compared to control, indicating absence of glial activation or proliferation, whereas rings were decreased in thickness around nontransduced neurons. Using GS as a marker of satellite glial cells, a significant increase in ring thickness was noted in nontransduced neurons after injection of AAV8-EGFP compared to control. Since this same group showed a decrease in ring thickness identified with GFAP, the significance of these changes is uncertain. We did not observe increased ATF3 expression, a marker for neuronal stress or injury [45], in sections from DRGs injected with vector compared to controls sections from DRGs injected with vehicle alone (**Fig. 6A, B**) or from naïve DRGs (22±1%), whereas the L5 SNL positive control sections exhibited strong ATF3 immunoreactivity (**Fig. 6A, B**). Similarly, Iba1 staining was not elevated even in areas with intense neuronal GFP expression (**Fig. 6A, B**), which suggests that the vectors did not activate resident microglia or causes infiltration of hematogenous macrophages into DRG. We also did not observe any positive cells immunolabeled by CD6, a pan-T cell marker, or CD8, a cytotoxic T-cell marker, compared to lymph node tissue as a positive control (data not shown). Specific staining for satellite glial cells (GFAP and GS) also clearly indicates that transduction and EGFP expression involves only neurons (**Fig. 6A**).

**Figure 7:**
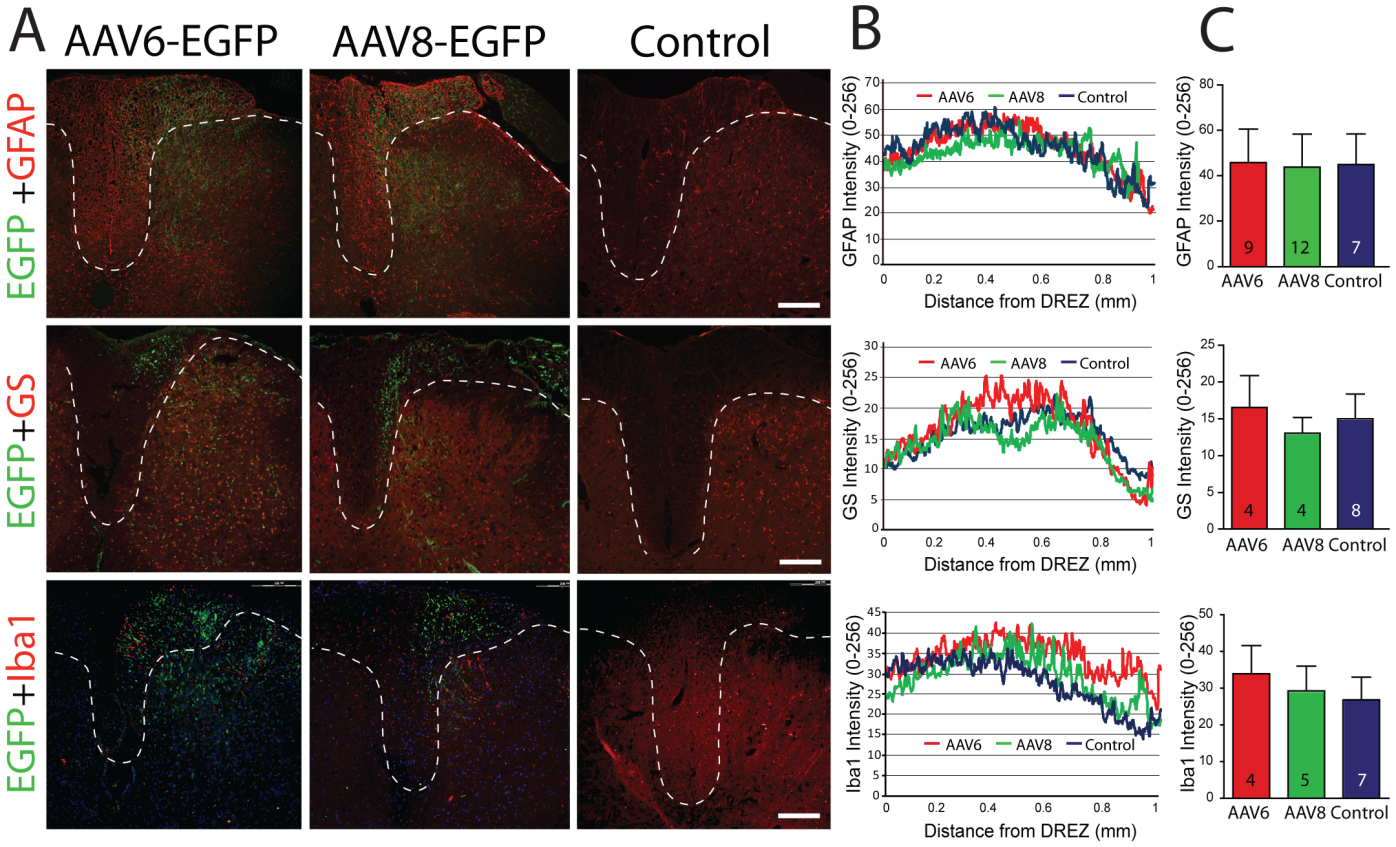
Identification of inflammatory response in dorsal horn (DH) of AAV injected rats. Representative sections (**A**) revealed no differences in astrocyte activation (GFAP and GS), or activation of resident microglia or infiltration of hematogenous macrophages (Iba1) between cord tissues from animals in which dorsal root ganglia were injected with AAV6-EGFP or AAV8-EGFP compared to saline injected control rats. Scale bar: 100 µm**.** Quantification of GFAP, GS, and Iba1 immunofluorescence (**B**) was done along lines overlaid from central canal to dorsal root entry zone (DREZ), producing traces of intensity. The averaged fluorescence intensities across the length of each trace (**C**) were similar for all groups. The number in each bar is the number of analyzed sections per group from at least 4 animals per group. Data are presented as mean ± SD. There were no differences between groups.

In spinal cord sections, GFAP-labeled astrocytes and Iba1-positive microglia/macrophages were observed in dorsal horns bilaterally (**Fig. 7A**), but glial proliferation or morphological evidence of activation was not observed for cord tissues from rats in which either AAV6-EGFP or AAV8-EGFP was injected into DRGs, compared to saline-injected controls (**Fig. 7A, B, C**). These findings indicate an absence of vector-induced histopathology in both the injected DRGs and associated cord levels.

### Behavioral Testing

**Figure 8:**
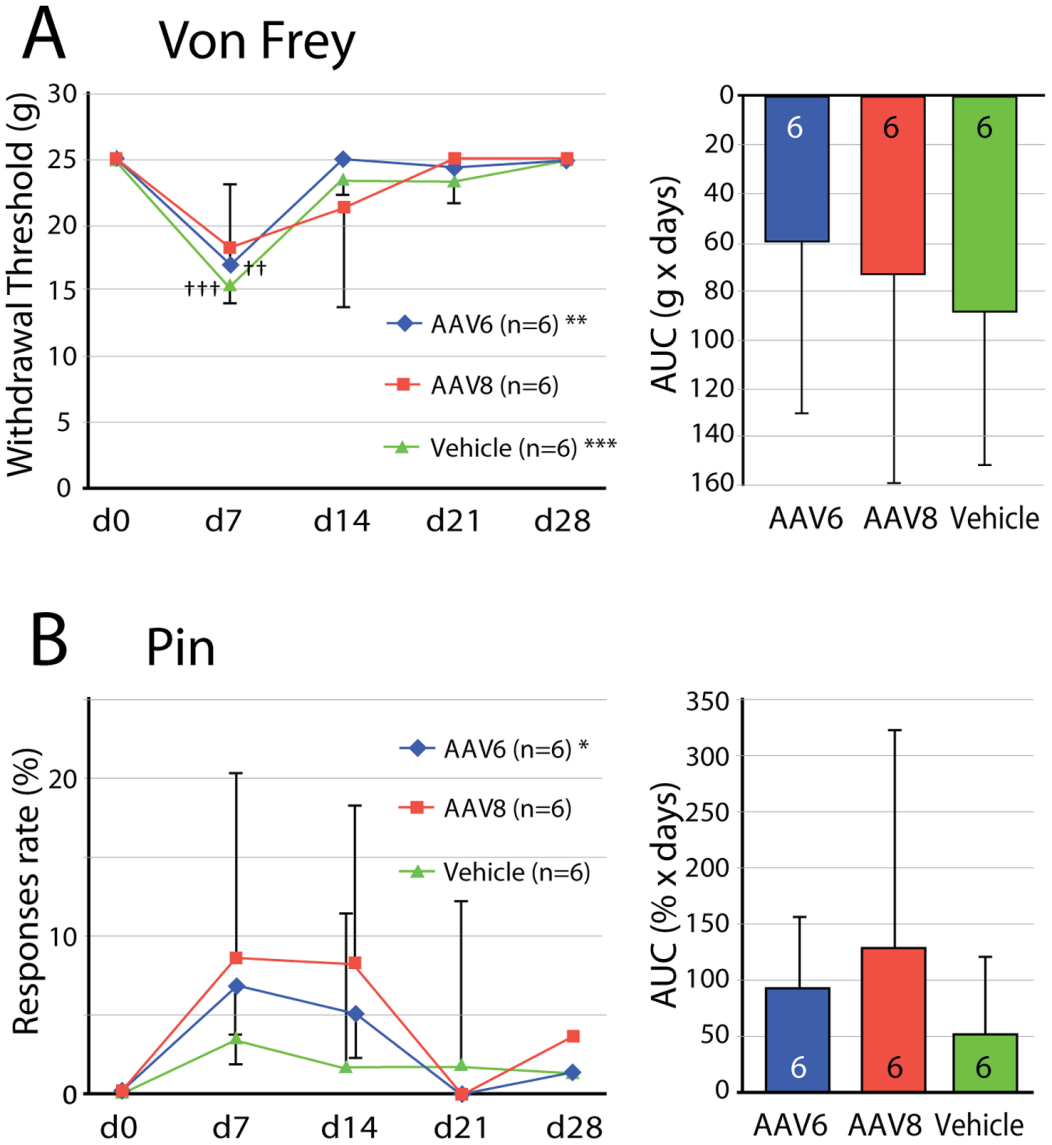
Pain behavior evaluation after intraganglionic AAVs. The response to innocuous punctate mechanical stimulation (von Frey, **A**) and hyperalgesia behavior after touch with a pin (Pin, **B**) following vehicle or vector injections evaluated across 4 weeks following vector injection. Left panels show the time course of testing performed upon the plantar surface of the right paw ipsilateral to injection of either vehicle (square), AAV6-EGFP (triangle) or AAV8-EGFP (diamond) into the fourth lumbar (L4) and L5 DRGs. Results are means ± SD (n  = 6 animals in each group). For ANOVA main effects, *p<0.05, **p<0.01, and ***p<0.001. For differences between the test day and baseline at day 0 (d0), ^††^p<0.01, ^†††^p<0.001. In the right panels, the time course data are analyzed using area under the curve (AUC) across the 4-week testing period for measures normalized to d0, allowing comparisons between vectors. Results are means ± SD. There were no differences between groups.

Since our previous experiments with AAV8 vector injections showed behavioral changes isolated to mechanosensation [2], we examined possible effects of the present vectors on this sensory modality. Sensitivity to cutaneous mechanical stimulation was evaluated prior to DRG injection of the vectors to obtain a baseline characterization, and was followed for 4 weeks after injection. Both vehicle and AAV6-EGFP produced a mild and transient mechanical allodynia state evident by von Frey mechanosensory threshold testing at 7 days after injection, while no change in withdrawal threshold was found in rats injected with AAV8-EGFP (**Fig. 8A**). Testing the rate of hyperalgesic response to noxious punctate mechanical stimulation (pin testing), which is an indicator of neuropathic pain [39] showed a main effect only for AAV6-EGFP, for which there was a transient initial rise in response rate (p<0.05). However, there were no significant differences from baseline on any day for either vector or for vehicle injection (**Fig. 8B**). AUC analysis of the behavioral data did not show differences between the two vectors and vehicle for von Frey or pin testing (**Fig. 8A, B**). There were no significant differences in the sensitivity of the rats for either vector 3-month post injection, compared to the baseline values, nor were there any differences between the ipsilateral and the contralateral paws (data not shown). The rats had normal appearance, level of activity, and weight gain.

## Discussion

We initiated this study as part of an overall program to develop optimal AAV vectors for gene delivery to primary sensory neurons, especially the nociceptive population that supports pain sensation. Since the DRG may have value as a target in gene therapy strategies for chronic pain, it was chosen as the primary injection site. Intraganglionic AAV vector injection has been shown to be feasible, safe, and efficient in targeting peripheral sensory neurons for local pain gene therapy [2], [3], [5], [46], and is superior to intrathecal delivery when restricting the spread of vector is desired [11], [47]. The two AAV vectors used in this study carried the identical genome with scAAV2 TR elements flanking the identical EGFP expression cassette, but differed by their capsid coats being either serotype 6 or 8. To provide the most valid comparison, the two vectors were produced with identical methods to yield preparations with comparably high purity and high content of full viral particles, and were applied *in vivo* by a standardized microinjection technique [2], [36]. With these factors controlled, any differences in neurotropism, transduction efficiency, persistence of transgene expression, or potential immunogenicity and neurotoxicity after intraganglionic delivery can be attributed to the different capsid properties.

We have shown that a single direct injection of AAV6 or AAV8 carrying EGFP into the lumbar DRG of adult rats achieves efficient and persistent EGFP expression selectively in the postmitotic primary sensory neurons and their central and peripheral processes. Both AAV6-EGFP and AAV8-EGFP exhibit strong neurotropism, without detectable transduction of non-neuronal cells. However, distinct neuronal transduction profiles are clearly demonstrated for these two different vectors. Overall, AAV6 transfers EGFP to the entire range of DRG neurons, while the population of AAV8-transduced neurons is shifted towards larger-sized neurons. AAV6-EGFP transduces approximately 50% of DRG neurons overall, with no difference between large neurons *versus* small- and medium-sized neurons, including those expressing the nociceptive markers CGRP and TrkA, and binding IB4 [48], [49], [50]. In contrast, AAV8-EGFP transduces approximately 30% of DRG neurons after direct injection, consistent with our previous report [2]. Our double immunofluorescence experiments confirm that the lower overall rate for AAV8-EGFP compared to AAV6-EGFP is attributable to the failure of AAV8-EGFP to transduce small neurons.

The central and peripheral termination patterns of EGFP-expressing transduced neurons reflect the distinct tropisms of AAV6-EGFP and AAV8-EGFP for DRG neuronal subgroups. AAV6-derived EGFP expression in central afferent fibers is present throughout the dorsal horn, especially in the superficial laminae that receive nociceptive input from unmyelinated (C-type) and thinly myelinated (Aδ-type) afferents, as well as thermal mechanoreceptive fibers [51]. This pattern is consistent with AAV6 targeting the full range of sensory neurons, including nociceptive and mechanoreceptive sensory DRG neurons. In contrast, AAV8-EGFP injected animals show minimal EGFP-labeled central projections to the superficial lamina, and instead show spinal EGFP mostly present in the dorsal column and the deeper dorsal horn (lamina III-V) where large myelinated sensory fibers terminate. A relatively greater population of transduced neurons project to the ventral horn after injection of AAV8-EGFP compared to AAV6-EGFP, consistent with AAV8-EGFP targeting fast-conducting proprioceptive neurons that primarily terminate in dorsal horn deeper lamina III-V and project into the ventral horn where they ramify and synapse with motoneurons [51]. Peripherally, EGFP signals are strong in the sciatic nerve in both AAV6-EGFP and AAV8-EGFP injected rats. However, whereas numerous EGFP-positive nerve terminals can be found in the plantar skin of the hind paw from AAV6-EGFP rats, AAV8-EGFP injection results in only rare transduced cutaneous fibers. This difference is also attributable to differential DRG neuron targeting by the vectors, since there is abundant skin innervation by small branching fibers that are susceptible to transduction by AAV6-EGFP, with relatively less dense innervation by myelinated fibers that AAV8-EGFP preferentially targets.

It has been reported that AAV6 has greater affinity for monocyte-derived dendritic cells (MoDCs) compared to other serotypes [52]. Because of this study, we performed immunohistochemistry evaluation of the effects of AAV6-EGFP and AAV8-EGFP vectors on cellular immunity, inflammatory response, and neurotoxicity, but we did not observe any inflammatory response, neurotoxicity, or CD6 and CD8 immunoreactive cells in the DRG sections. Recent studies have shown that AAV epitopes recognized by CD8-positive T-cells and antibodies in humans and rodents are highly conserved among the many AAV serotypes, resulting in humoral and T cell-mediated immunity against different AAV serotypes [53], [54], [55], [56]. Evidently, the AAV vectors we tested do not induce strong adaptive immune responses that would reduce the efficacy and stability of *in vivo* gene transfer [57].

Our findings regarding the preferential transduction of large neurons by AAV8-EGFP are consistent with observations following intraganglionic or IT delivery of AAV8 [2], [13], [24]. However, our observations are inconsistent with other prior findings. Intrasciatic AAV8 preferentially transduces Schwann cells [58], whereas we see only neuronal transduction after DRG injection. Intrathecal (IT) AAV6 is ineffective at transducing DRG neurons in one report [59], although another [11] finds that IT and sciatic nerve injection are effective in transducing DRG neurons, including the small sized population, similar to our findings after DRG injection. Another report in which various AAV vectors were compared for gene transfer by intraganglionic delivery, showed that AAV6 and AAV8 did not differ in transduction efficiency at 2 weeks, but that AAV6 induces only short term transgene expression [3]. Tropism for specific neuronal subtypes, induction of inflammatory indicators, and behavioral effect were not explored in that study. Numerous factors may complicate direct comparisons of the results from different laboratories. In addition to the importance of capsid sequences, AAV *in vivo* performance is also affected by many other factors including the delivery strategy and potential local trauma, the transgene promoter (expression instability because of promoter methylation), transgene regulatory sequences (transcriptional enhancing intron and mRNA stable Woodchuck hepatitis virus posttranscriptional regulatory element [60], [61], [62]), the choice of single-strand or self-complementary AAV [63], dose-dependent toxicity [64], recipient species and age effects on transduction efficiency and immune response to AAV vectors, presence of pre-existing antibodies to the vector, and variable potency of vectors using different production and titering methods. Technical details of the vector preparation may affect their performance [28], so the divergent transduction patterns between our study and the other reports may also result in part from methodological variations. We have been successful using Optiprep discontinuous gradient ultracentrifugation to separate empty from full viral particles. Empty particles can inhibit transduction efficiency of full particles and induce innate immune responses [57], [65], [66], which highlights the importance of controlling these factors in comparative studies.

In conclusion, although both self-complementary AAV6 and AAV8 are both effective and safe vectors for transgene delivery to primary sensory neurons, there are critical distinctions. Intraganglionic delivery of AAV6 transfers genes to the full range of DRG neurons and their terminals, including the nociceptive subpopulation, whereas AAV8 is comparatively restricted to large neurons. This results in an overall higher transduction rate for AAV6 compared to AAV8. Other favorable features of intraganglionic AAV6 delivery for gene transfer in the peripheral sensory nervous system are long-term stable transgene expression (up to 3 months), the lack of apparent vector-induced neurotoxicity or inflammation in DRGs and spinal cord, and minimal sensory changes in treated animals. These results will aid future investigation in the choice of serotype for specific gene delivery applications, and provide a basis for comparison when these and other serotypes are used in large animal models.
