# Correction: Intraganglionic AAV6 Results in Efficient and Long-Term Gene Transfer to Peripheral Sensory Nervous System in Adult Rats

**DOI:** 10.1371/journal.pone.0117887

**Published:** 2015-02-10

**Authors:** The PLOS ONE Staff

There are errors in the Funding section. The correct funding information is as follows: This study was funded in part by the VA Rehabilitation Research and Development grant 3690-03 (http://www.research.va.gov/). Lejla Ferhatovic was supported by the Croatian Foundation for Science (HRZZ) grant no. 02.05./28. The funders had no role in study design, data collection and analysis, decision to publish, or preparation of the manuscript.

## References

[1] Yu H, Fischer G, Ferhatovic L, Fan F, Light AR, et al (2013) Intraganglionic AAV6 Results in Efficient and Long-Term Gene Transfer to Peripheral Sensory Nervous System in Adult Rats. PLoS ONE 8(4): e61266 doi:10.1371/journal.pone.0061266 23613824 10.1371/journal.pone.0061266 PMC3628918

